# Biocontrol of Soft Rot *Dickeya* and *Pectobacterium* Pathogens by Broad-Spectrum Antagonistic Bacteria within *Paenibacillus polymyxa* Complex

**DOI:** 10.3390/microorganisms11040817

**Published:** 2023-03-23

**Authors:** Afsana Hossain, Md. Arshad Ali, Li Lin, Jinyan Luo, Yuxin You, Md. Mahidul Islam Masum, Yugen Jiang, Yanli Wang, Bin Li, Qianli An

**Affiliations:** 1 State Key Laboratory of Rice Biology and Breeding, Ministry of Agriculture Key Laboratory of Molecular Biology of Crop Pathogens and Insects, Zhejiang Province Key Laboratory of Biology of Crop Pathogens and Insects, Institute of Biotechnology, Zhejiang University, Hangzhou 310058, China; 2 Department of Plant Pathology, Bangabandhu Sheikh Mujibur Rahman Agricultural University, Gazipur 1706, Bangladesh; 3 Sugarcane Research Institute, Guangxi Academy of Agricultural Sciences, Nanning 530007, China; 4 Department of Plant Quarantine, Shanghai Extension and Service Center of Agriculture Technology, Shanghai 201103, China; 5 Agricultural Technology Extension Center of Fuyang District, Hangzhou 311400, China; 6 State Key Laboratory for Quality and Safety of Agro-Products, Institute of Plant Protection and Microbiology, Zhejiang Academy of Agricultural Sciences, Hangzhou 310021, China

**Keywords:** biocontrol, soft rot disease, cyclic lipopeptides, plant growth-promoting bacteria

## Abstract

Polymyxin-producing bacteria within the *Paenibacillus polymyxa* complex have broad-spectrum activities against fungi and bacteria. Their antibacterial activities against soft rot *Dickeya* and *Pectobacterium* phytopathogens containing multiple polymyxin-resistant genes were not clear. Here, we selected nine strains within the *P. polymyxa* complex having broad-spectrum antagonistic activities against phytopathogenic fungi and a polymyxin-resistant *D. dadantii* strain causing stem and root rot disease of sweet potato and did antagonistic assays on nutrient agar and sweet potato tuber slices. These strains within the *P. polymyxa* complex showed clear antagonistic activities against *D. dadantii* in vitro and in vivo. The most effective antagonistic strain *P. polymyxa* ShX301 showed broad-spectrum antagonistic activities against all the test *Dickeya* and *Pectobacterium* strains, completely eliminated *D. dadantii* from sweet potato seed tubers, and promoted the growth of sweet potato seedlings. Cell-free culture filtrate of *P. polymyxa* ShX301 inhibited *D. dadantii* growth, swimming motility, and biofilm formation and disrupted *D. dadantii* plasma membranes, releasing nucleic acids and proteins. Multiple lipopeptides produced by *P. polymyxa* ShX301 may play a major role in the bactericidal and bacteriostatic actions. This study clarifies that the antimicrobial spectrum of polymyxin-producing bacteria within the *P. polymyxa* complex includes the polymyxin-resistant *Dickeya* and *Pectobacterium* phytopathogens and strengthens the fact that bacteria within the *P. polymyxa* complex have high probability of being effective biocontrol agents and plant growth promoters.

## 1. Introduction

Gram-negative *Pectobacterium* and *Dickeya* bacteria produce multiple pectinases and cause destructive soft rot diseases of numerous crops and ornamental plants and great economic losses worldwide [1,2]. While the worldwide distribution of soft rot *Pectobacterium* was well-known, soft rot *Dickeya* was previously described as being present mainly in tropical and subtropical regions but is expanding its global distribution. For example, *Dickeya* spp. associated with blackleg of potato and foot rot of rice spread rapidly in Europe as far north as Finland near the Arctic Circle [3,4] and in China from South China to Northeast China [5]. Importantly, soft rot *Dickeya* is threatening staple food crops, including potato [3,6], rice [7], maize [8], sweet potato [9,10], and banana [11,12].

Management of soft rot diseases on crops has not been successful due to the lack of resistant crop varieties and the end of large-scale use of effective antibiotics for the risk of introducing resistance to bacterial pathogens in humans or animals [1]. Biological control is an alternative to the breeding of resistant crops and chemical control the diseases and has been increasingly tested to control *Pectobacterium* and *Dickeya* pathogens, particularly using bacteriophages with restricted bacterial hosts [13] and broad-spectrum antagonistic bacteria [14,15].

Gram-positive *Bacillus* and *Paenibacillus* bacteria, which produce multiple antimicrobial compounds against broad phytopathogens and produce endospores resistant to heating and dryness for the formulation of stable products, are high-profile biological control agents [16,17,18]. *Paenibacillus polymyxa* is well-known for its production of the antibiotic polymyxins, which are cyclic lipopeptides and used as last-resort agents against notorious Gram-negative multi-drug resistant human pathogens, including *Acinetobacter baumannii*, *Pseudomonas aeruginosa*, and *Klebsiella pneumoniae* [19]. *P. polymyxa* forms a monophyletic species complex with *P. peoriae*, *P. kribbensis*, *P. ottowii*, *P. brasilensis*, *P. terrae*, and “*P. maysiensis*,” most of which also produce the cyclic lipopeptides-fusaricidins against broad-spectrum fungi, oomycetes, and Gram-positive bacteria, including important phytopathogens [16,20].

Polymyxins are a group of decapeptides containing five to six residues of the nonproteogenic amino acid 2,4-diaminobutyric acid, resulting in a high positive charge density. Polymyxins bind to the lipid A component of lipopolysaccharides on the outer membrane of Gram-negative bacteria and disrupt the outer membrane and then permeabilize and disrupt the inner membrane [16,21]. The general mechanism of bacterial resistance to polymyxins involves modification of lipid A, such as the addition of 4-aminoarabinose by the *arnB* operon products, the addition of phosphoethanolamine by EptA, hydroxylation of lipid A acyl chains by LpxO, and acylation or deacylation of lipid A by PagP or PagL [22], thereby reducing the net negative charge and polymyxin-binding affinity [16,21]. In Gram-positive bacteria, resistance to antimicrobial peptides occurs in the esterification of phosphate with alanine of teichoic acids by the *dltXABCD* operon products [23]. In all the sequenced genomes of *Dickeya* and *Pectobacterium*, not only *arnB* operon and *eptA* are present similar to other enterobacteria, but also *dlt* operon in Gram-positive bacteria is present [24]. These genes confer resistance to polymyxins.

Polymyxin-resistant *Dickeya* and *Pectobacterium* may resist the antagonistic activity from polymyxin-producing strains within the *P. polymyxa* complex. However, previous studies have not intentionally tested this hypothesis. Here, we tested if the antagonism of the *P. polymyxa* complex is negative on the polymyxin-resistant *Dickeya* and *Pectobacterium* and see if the antagonistic spectrum of the *P. polymyxa* complex can extend to the polymyxin-resistant *Dickeya* and *Pectobacterium*. We selected strains within the *P. polymyxa* complex previously screened out for their broad-spectrum antagonistic activities against phytopathogenic fungi [20,25] to test their antagonistic activities against *Dickeya* and *Pectobacterium*. A polymyxin-resistant *D. dadantii* strain was used for the first-round screening. Contrary to the hypothesis, all the test strains within the *P. polymyxa* complex showed clear antagonistic activities against *D. dadantii,* and the most effective strain *P. polymyxa* ShX301 showed antagonism to all the tested *Dickeya* and *Pectobacterium* strains. We further studied the antibacterial profiles of *P. polymyxa* ShX301 and showed its application potential to control diseases in fields.

## 2. Materials and Methods

### 2.1. Bacterial Strains and Media

Three strains isolated from the rhizosphere soils of cotton plants [25] and six strains isolated from legume nodules [20] were recently screened out for their broad-spectrum antagonistic activities against phytopathogenic fungi. *Dickeya dadantii* strain CZ1501 is a causal agent of the bacterial stem, and the root disease of sweet potato occurred in Hangzhou, Zhejiang province, China. Its whole genome sequence (GenBank accession number MPDL00000000.1) contains the *arnB*, *eptA,* and *dlt* operons for resistance of polymyxins. It can grow in nutrient broth with 2 μg·mL^−1^ of polymyxin B sulfate. Six other *Dickeya* and two *Pectobacterium* strains were obtained from culture collections (Table 1).

Bacterial strains were cultured in nutrient broth (10 g tryptone, 3 g beef extract, 2.5 g glucose, 5 g NaCl per liter, pH 7.0) or on nutrient agar (nutrient broth with 20 g agar per liter). Bacterial cultures in nutrient broth were washed with sterile water and adjusted to an optical density of 0.6 at 600 nm (OD600); the cell number of the suspension was counted by serial dilution and plating on nutrient agar. Bacterial suspensions were finally adjusted to the concentration of 1 × 10^8^ CFU·mL^−1^ for use.

### 2.2. In Vitro and In Vivo Screening of Antagonistic Paenibacillus Strains

*Paenibacillus* strains against *D. dadantii* CZ1501 were screened using the in vitro overly culture assay on nutrient agar plates and the in vivo tuber slice assay as previously described [15]. Antagonistic activities of the *P. polymyxa* stain ShX301 were further tested on other *Dickeya* and *Pectobacterium* strains (Table 1) using the in vitro overlay culture assay on both the nutrient agar and the M9 minimal agar [15]. The in vitro inhibition rate (%) on target strains by *Paenibacillus* was calculated by [1-(diameter of *Paenibacillus* colony/diameter of inhibition zone)] × 100. The in vivo inhibition rate (%) was calculated by [1-(diameter of maceration zone by *D. dadantii* with *Paenibacillus*/diameter of maceration zone by *D. dadantii* without *Paenibacillus*] × 100.

### 2.3. In Vivo Biocontrol of D. dadantii by P. polymyxa ShX301

The biocontrol potential of the *P. polymyxa* stain ShX301 was determined using the in vivo assay with sweet potato seed tubers as previously described [15]. Surface-sterilized seed tubers were immersed in sterile distilled water (control), *D. dadantii* suspension (1 × 10^8^ CFU·mL^−1^), *P. polymyxa* suspension (1 × 10^8^ CFU·mL^−1^), or both *D. dadantii* and *P. polymyxa* at 1 × 10^8^ CFU·mL^−1^ for 4 h at 25 °C and then kept under 28 °C, a photoperiod of 12-h light and 12-h dark, and 80% relative humidity for 21 d.

### 2.4. Antibacterial Activities of Cell-Free Culture Supernatant (CFCS) of P. polymyxa ShX301 against D. dadantii

*P. polymyxa* ShX301 was cultured in nutrient broth at 30 °C with shaking at 200 rpm for 48 h and adjusted to about 1 × 10^8^ CFU·mL^−1^ with the nutrient broth. CFCS of the bacterial suspension was obtained by centrifugation and filtering through a 0.22-µm filter and confirmed by incubating 100 µL of the CFCS on nutrient agar at 30 °C for 48 h.

CFCS effects on *D. dadantii* growth in wells of sterile polystyrene flat-bottom 96-well microplates, biofilm formation on the surface of the microplate wells, and swimming motility in 0.3% (*w/v*) agar were determined as previously described [15].

CFCS effects on *D. dadantii* cell integrity were observed by transmission electron microscopy on *D. dadantii* cells (1 × 10^8^ CFU·mL^−1^) at mid-exponential phase grown with CFCS (50% volume) for 4 h. *D. dadantii* cells were washed twice with 0.1 mol·L^−1^ phosphate buffer (pH 7) and fixed in 2.5% (*v/v*) glutaraldehyde in the phosphate buffer at 4 °C overnight. After washing with the phosphate buffer, *D. dadantii* cells were fixed in 1% (*w/v*) OsO_4_ dissolved in the phosphate buffer for 1 h at room temperature. *D. dadantii* cells were then washed with distilled water and dehydrated by a graded series of ethanol. Dehydrated *D. dadantii* cells were infiltrated by Spurr’s resin at room temperature and embedded in Spurr’s resin at 70 °C for 9 h. Ultrathin sections were cut with glass knives on an ultramicrotome (Reichert-Jung, Vienna, Austria), collected on copper grids, stained with uranyl acetate and lead citrate, and observed with the JEM-1230 transmission electron microscope (JEOL, Tokyo, Japan).

CFCS effects on *D. dadantii* cell integrity were determined by measuring the release of nucleic acids (absorbance at 260 nm, OD260) and proteins (absorbance at 280 nm, OD280) from *D. dadantii* cells (1 × 10^8^ CFU·mL^−1^) at mid-exponential phase grown with CFCS (50% volume) for 4 h as previously described [15].

### 2.5. Detection of Lipopeptides Produced by P. polymyxa ShX301

Lipopeptides in the CFCS of *P. polymyxa* ShX301 were detected by matrix-assisted laser desorption/ionization coupled with time-of-flight mass spectrometry (MALDI-TOF MS) as previously described [15].

### 2.6. Statistical Analysis

Data were subjected to a one-way analysis of variance, and means were compared by Duncan’s multiple range test using the SPSS software version 16 (SPSS, Chicago, IL, USA). The significance was set at *p* < 0.05.

## 3. Results

### 3.1. Strains within the P. polymyxa Complex Inhibited D. dadantii Growth and Maceration of Sweet Potato Tuber Slices

All tested strains within the *P. polymyxa* complex inhibited *D. dadantii* growth in the nutrient agar and generated clearing zones around the *Paenibacillus* colonies. *P. polymyxa* ShX301 generated relatively larger clearing zones and showed a significantly higher rate (53%) of in vitro inhibition of *D. dadantii* growth than other *Paenibacillus* strains did.

*D. dadantii* CZ1501 degraded plant cell walls and generated maceration zones about 36 mm in diameter at 24 h after inoculation into the sweet potato tuber slices. All tested *Paenibacillus* strains did not macerate the tuber tissues but inhibited the maceration from *D. dadantii*. The in vivo inhibition of the maceration from *D. dadantii* was consistent with the in vitro inhibition of *D. dadantii* growth in the nutrient agar (Table 2). *P. polymyxa* ShX301 showed the highest in vivo inhibition rate (80%) at the *D. dadantii* maceration of sweet potato tuber slices. Therefore, *P. polymyxa* ShX301 was the most effective strain against *D. dadantii* and was used for further analyses.

### 3.2. P. polymyxa ShX301 Showed Broad-Spectrum Antagonistic Activities against Dickeya and Pectobacterium

*P. polymyxa* ShX301 showed antagonistic activities against all the tested *Dickeya* and *Pectobacterium* strains on the nutrient agar and the M9 minimal agar (Table 3). It showed more potent antagonistic activities on the M9 minimal agar than on the nutrient agar.

### 3.3. P. polymyxa ShX301 Protected Seed Tubers and Promoted Seedling Growth

**Figure 1:**
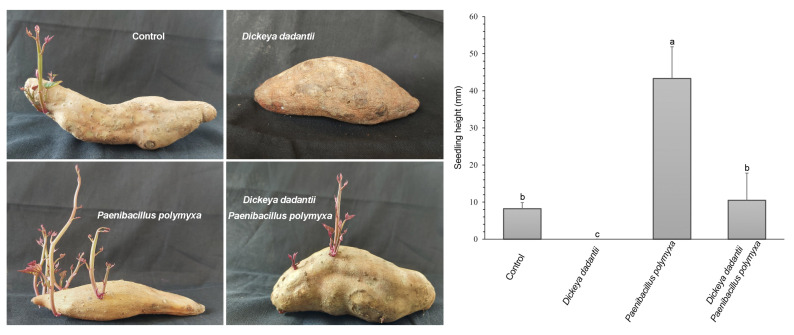
Sweet potato seed tubers and seedlings at 21 d after inoculation with water, *Dickeya dadantii* CZ1501, *Paenibacillus polymyxa* ShX301, or both *D. dadantii* and *P. polymyxa*. Vertical bars represent the standard errors of mean values (*n* = 6). Different letters on the bars indicate a significant difference between the treatments at *p* < 0.05.

Seed tubers inoculated with only *D. dadantii* did not germinate and were rotten; *D. dadantii* was isolated from the rotten seed tubers at 21 d after inoculation. In contrast, seed tubers inoculated with only *P. polymyxa* ShX301 germinated and grew seedlings significantly higher than the control seedlings. Seed tubers inoculated with *D. dadantii* along with *P. polymyxa* ShX301 germinated and grew seedlings slightly higher than the control seedlings (Figure 1). *D. dadantii* was not isolated from the seedlings inoculated with *P. polymyxa* ShX301.

### 3.4. CFCS of P. polymyxa ShX301 Inhibited D. dadantii Growth, Biofilm Formation, and Swimming Motility

**Figure 2:**
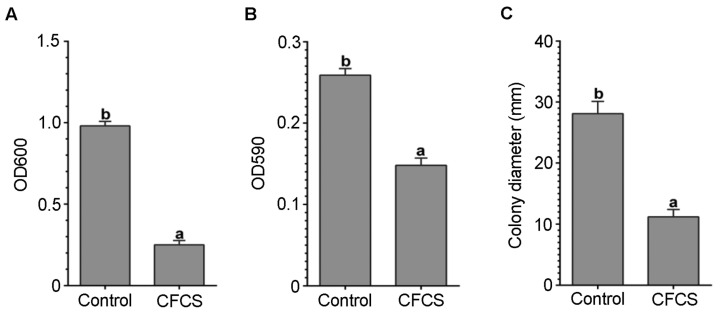
Antibacterial activities of cell-free culture supernatant (CFCS) of *Paenibacillus polymyxa* ShX301 against *Dickeya dadantii* CZ1501. (**A**) *D. dadantii* growth in nutrient broth in microplate wells determined by optical density at 600 nm (OD600). (**B**) Biofilm formed on the surface of microplate wells by *D. dadantii* during 24-h incubation determined by crystal violet absorbance at 590 nm (OD590). (**C**) Diameter of *D. dadantii* colonies formed on 0.3% (*w/v*) agar via swimming. Vertical bars represent the standard errors of mean values (*n* = 6). Different letters on the bars indicate a significant difference between the treatments at *p* < 0.05.

*D. dadantii* growth in nutrient broth in the microplate wells and biofilm formation on the surface of the microplate wells were significantly inhibited when the CFCS of *P. polymyxa* ShX301 was present at 50% volume (Figure 2A,B). *D. dadantii* CZ1501 swam via flagella in 0.3% (*w/v*) agar and formed haloes about 28 mm in diameter after 48 h. The swimming motility of *D. dadantii* was almost lost and formed haloes about 11 mm in diameter when the CFCS was present at 50% volume (Figure 2C).

### 3.5. CFCS of P. polymyxa ShX301 Breached D. dadantii Cells

**Figure 3:**
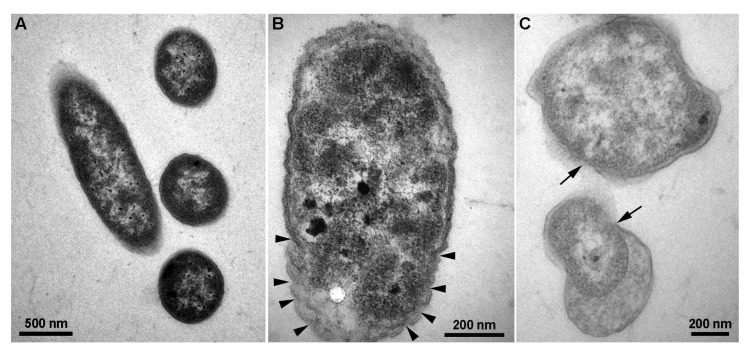
Transmission electron micrographs show cells of *Dickeya dadantii* CZ 1501. (**A**) Control cells with intact cell envelopes and electron-dense cytoplasm. (**B**,**C**) Damaged *D. dadantii* cells with convoluted cell envelopes (arrowheads), breached cell envelopes (arrows), and cleared cytoplasm under the cell-free culture supernatant of *Paenibacillus polymyxa* ShX301.

Transmission electron microscopy showed that control *D. dadantii* cell envelopes were intact and enclosed electron-dense cytoplasm (Figure 3A) while cell envelopes were convoluted (Figure 3B) or beached and cytoplasm was clearing (Figure 3C) when the CFCS of *P. polymyxa* ShX301 were present for 4 h. Under the CFCS of *P. polymyxa* ShX301, damage to *D. dadantii* cells was also indicated by the release of nucleic acids (128 µg·mL^−1^) and proteins (2.75 mg·mL^−1^) determined by the increases of the OD260 value (0.33) and OD280 value (0.31), respectively.

### 3.6. P. polymyxa ShX301 Produced Multiple Lipopeptides

**Figure 4:**
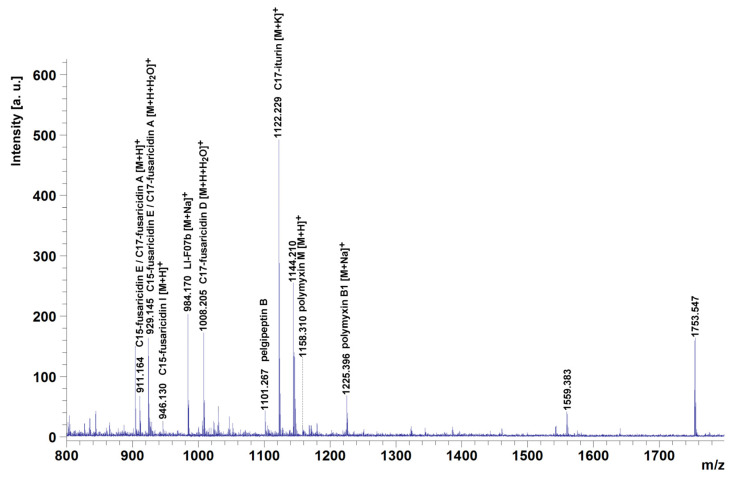
MALDI-TOF mass spectrum of cell-free culture supernatant of *Paenibacillus polymyxa* ShX301 in the range of *m*/*z* 800–1800.

MALDI-TOF MS detected lipopeptides which appeared in a protonated form [M+H]^+^ and alkali adducts, such as [M+Na]^+^ and [M+K]^+^. Fusaricidins also appeared as open-chain products [M+H+H_2_O]^+^ [26,27]. MALDI-TOF MS detected eight distinct clusters of mass peaks (*m*/*z* 802–865, *m*/*z* 904–946, *m*/*z* 984–1052, *m*/*z* 1101, *m*/*z* 1122, *m*/*z* 1144–1225, *m*/*z* 1542–1559 and *m*/*z* 1753) in the range of *m*/*z* 800–1800 from CFCS of *P. polymyxa* ShX301 (Figure 4). The mass peaks *m*/*z* 904–946 and *m*/*z* 984–1052 were in the *m*/*z* range for fusaricidins [26,27]. The mass peak *m*/*z* 1144–1225 was in the *m*/*z* range for polymyxins. The mass peak *m*/*z* 1101 may correspond to pelgipeptin B [28,29]. The mass peak *m*/*z* 1122 was close to *m*/*z* 1122.6 [M+K]^+^ of C17-iturin [30]. The mass peaks *m*/*z* 802–865, *m*/*z* 1559, and *m*/*z* 1753 were not identified.

## 4. Discussion

Contrary to the hypothesis that polymyxin-resistant *Dickeya* and *Pectobacterium* may resist the antagonism from polymyxin-producing strains within the *P. polymyxa* complex, all the test strains within the *P. polymyxa* complex, which have broad-spectrum antagonistic activities against fungal phytopathogens [20,25], showed clear antagonistic activities against the polymyxin-resistant *D. dadantii* strain CZ1501. Moreover, the most effective strain *P. polymyxa* ShX301 showed clear antagonistic activities against all the tested *Dickeya* and *Pectobacterium* strains. The extents of the in vitro inhibition of *D. dadantii* growth in nutrient agar and the in vivo inhibition of *D. dadantii* maceration in sweet potato tuber slices are consistent. *P. polymyxa* ShX301 showed more potent antibacterial activities against *Dickeya* and *Pectobacterium* strains on the nutrient-limited minimal medium than on the nutrient-rich medium. Thus, it may inhibit soft rot pathogens in nutrient-limited niches, such as soils, leaf surfaces, and seed tuber surfaces.

CFCS of *P. polymyxa* ShX301 inhibited *D. dadantii* growth, indicating that *P. polymyxa* released antibacterial compounds into the CFCS. The antibacterial compounds in the CFCS appeared to distort and breach *D. dadantii* cell envelopes, particularly plasma membranes, leading to the release of cell contents, such as nucleic acids and proteins, which were revealed by transmission electron microscopy and spectrophotometry. Disrupting plasma membranes indicates the involvement of amphiphilic lipopeptides in the antibacterial mechanisms of *P. polymyxa*.

MALDI-TOF-MS revealed that *P. polymyxa* ShX301 produced fusaricidins, polymyxins, and pelgipeptin-like and iturin-like lipopeptides. Fusaricidins have potent antimicrobial activities against fungi and Gram-positive bacteria but no or weak activity against Gram-negative bacteria [21,31] and thus may not contribute to the cytotoxic action on *D. dadantii*. Polymyxins consist of up to 30 closely related lipopeptides against Gram-negative bacteria [19], while *Dickeya* and *Pectobacterium* have evolved multiple pathways (*arnB*, *eptA,* and *dlt* operons) to resist polymyxins [24]. However, polymyxin P was found to inhibit the growth of *Pectobacterium carotovorum* (formerly *Erwinia carotovora*) [32]. Here, although *D. dadantii* CZ1501 resists 2 μg·mL^−1^ of polymyxin B, multiple polymyxins produced by *P. polymyxa* ShX301 may accumulate to a locally high concentration, contributing to the cytotoxic action on the damage of *D. dadantii* plasma membrane and the inhibition of *D. dadantii* growth in vitro, in vivo, and in planta. Pelgipeptins produced by *Paenibacillus* have broad-spectrum activities against Gram-positive and Gram-negative bacteria and fungi, including phytopathogens [28,29,33,34]. Iturins, which are produced by *Bacillus* and have potent antifungal activity and limited antibacterial activity [21], have also been found to inhibit the growth of phytopathogenic bacteria, including *P. carotovorum* [35]. The pelgipeptin-like and iturin-like lipopeptides from *P. polymyxa* ShX301 may also contribute to the cytotoxic action on *D. dadantii*.

Flagella-mediated motility and biofilm formation facilitate *D. dadantii* to colonize plant surface, intercellular spaces, and xylem vessels and complete disease cycles [36,37,38,39]. CFCS of *P. polymyxa* ShX301 inhibited *D. dadantii* swimming motility and biofilm formation. Fusaricidins and polymyxins from *P. polymyxa* may not play a major role in cytotoxic action on *D. dadantii* but may act as biosurfactants to inhibit surface attachment and biofilm formation [40], thus reducing *D. dadantii* infection and increasing *D. dadantii* susceptibility to cytotoxic lipopeptides. Multiple lipopeptides produced by *P. polymyxa* ShX301 play a major role in bactericidal and bacteriostatic actions on the phytopathogens.

From the view of plant-microbe interactions, plants can induce the expression of polymyxin and fusaricidin biosynthesis genes in *P. polymyxa* [41], while lipopeptides such as fusaricidins can induce plant systemic resistance to fungal and bacterial pathogens [42,43]. Notably, *P. polymyxa* ShX301 completely eliminated *D. dadantii* from sweet potato seed tubers at 21 d after the equivalent inoculation of the two bacteria. Bactericidal and bacteriostatic actions and induced plant systemic resistance mediated by multiple lipopeptides may play together to eliminate *D. dadantii*. The latent infection of seed tubers by *Dickeya* and *Pectobacterium* is a major source of the soft rot diseases of potato and sweet potato plants [2,44]. *P. polymyxa* ShX301 has shown the potential to control soft rot diseases from seed tuber-borne pathogens. Moreover, inoculating *P. polymyxa* ShX301 alone to sweet potato seed tubers promoted the growth of sweet potato seedlings. Likewise, *P. polymyxa* ShX301 promoted the growth of cotton seedlings and suppressed the soil-borne fungal pathogen *Verticillium dahlia* and the Verticillium wilt disease of cotton seedlings [25]. Therefore, *P. polymyxa* ShX301 is a promising biocontrol agent and plant growth promoter for future application in fields and study of the underlying mechanisms of the broad-spectrum antagonism and mutually beneficial interactions with plants.

## 5. Conclusions

This study clarifies that the antimicrobial spectrum of polymyxin-producing bacteria within the *P. polymyxa* complex includes the polymyxin-resistant soft rot *Dickeya* and *Pectobacterium* pathogens and strengthens the fact that bacteria within the *P. polymyxa* complex have high probability of being effective biocontrol agents and plant growth promoters. *P. polymyxa* ShX301 will be used to clarify the biocontrol mechanism of the *P. polymyxa* complex against broad-spectrum phytopathogens, including polymyxin-resistant *Dickeya* and *Pectobacterium* and to control plant diseases in fields.

## Figures and Tables

**Table 1:** Bacterial strains used in this study.

Organism	Isolation Source
*Paenibacillus polymyxa* ShX301	Cotton rhizosphere
*P. polymyxa* ShX302	Cotton rhizosphere
*P. polymyxa* ShX303	Cotton rhizosphere
*P. polymyxa* RP31	Black locust nodule
*P. peoriae* RP20	Black locust nodule
*P. peoriae* RP51	Black locust nodule
*P. peoriae* RP62	Black locust nodule
*P. peoriae* CFCC 1854	*Dendrolobium triangulare* nodule
*P. kribbensis* CFCC 1865	*Ormosia semicastrata* nodule
*Dickeya dadantii* CZ1501	Sweet potato
*D. dadantii* NCPPB 898^T^	*Pelargonium capitatum*
*D. solani* NCPPB 4479	Potato
*D. dianthicola* NCPPB 3534	Potato
*D. fangzhongdai* CGMCC 1.15464^T^	*Pyrus pyrifolia*
*D. chrysanthemi* NCPPB 402^T^	Chrysanthemum
*D. oryzae* ACCC 61554^T^	Rice
*Pectobacterium carotovorum* CFCC 10814^T^	Potato
*Pectobacterium cacticida* CFCC 10813^T^	Cactus

**Table 2:** Inhibition of *Dickeya dadantii* by strains within *Paenibacillus polymyxa* complex based on in vitro overlay culture assay and in vivo tuber slice assay.

*Paenibacillus* Strains	In Vitro Inhibition Rate (%)	In Vivo Inhibition Rate (%)
*P. polymyxa* ShX301	53 ± 4 ^a,^*	80 ± 2 ^a,^*
*P. polymyxa* ShX302	39 ± 3 ^bc^	57 ± 1 ^c^
*P. polymyxa* ShX303	44 ± 3 ^ab^	70 ± 1 ^b^
*P. polymyxa* RP 31	42 ± 1 ^ab^	67 ± 2 ^b^
*P. peoriae* RP 51	37 ± 5 ^bc^	57 ± 2 ^c^
*P. peoriae* RP 20	16 ± 3 ^e^	33 ± 2 ^f^
*P. peoriae* RP 62	13 ± 4 ^e^	20 ± 1 ^g^
*P. peoriae* CFCC 1854	22 ± 3 ^de^	38 ± 2 ^e^
*P. kribbensis* CFCC 1865	30 ± 2 ^cd^	48 ±2 ^d^

* The different letters following the mean value ± standard error in the same column indicate a significant difference between the treatments at *p* < 0.05.

**Table 3:** Inhibition of *Dickeya* and *Pectobacterium* strains by *P. polymyxa* ShX301 based on in vitro overlay culture assays on nutrient agar and M9 minimal agar.

*Dickeya* and *Pectobacterium*	Inhibition Rate (%)	
	Nutrient Agar	M9 Agar
*D. dadantii* CZ1501	53 ± 3 ^a,^*	74 ± 2 ^b,^*
*D. dadantii* NCPPB 898^T^	50 ± 2 ^a^	70 ± 3 ^b^
*D. solani* NCPPB 4479	45 ± 2 ^a^	65 ± 2 ^b^
*D. dianthicola* NCPPB 3530	54 ± 3 ^a^	72 ± 2 ^b^
*D. fangzhongdai* CGMCC 1.15464^T^	48 ± 1 ^a^	69 ± 2 ^b^
*D. chrysanthemi* NCPPB 402^T^	53 ± 1 ^a^	72 ± 1 ^b^
*D. zeae *NCPPB 3531	46 ± 3 ^a^	64 ± 1 ^b^
*P. carotovorum* CFCC 10814^T^	54 ± 2 ^a^	72 ± 3 ^b^
*P. cacticida* CFCC 10813^T^	52 ± 2 ^a^	70 ± 2 ^b^

* The letters a and b following the mean value ± standard error in the same row indicate a significant difference between the treatments at *p* < 0.05.

## Data Availability

Not applicable.
